# Spectrum of Trained Innate Immunity Induced by Low-Virulence *Candida* Species against Lethal Polymicrobial Intra-abdominal Infection

**DOI:** 10.1128/IAI.00348-19

**Published:** 2019-07-23

**Authors:** Elizabeth A. Lilly, Junko Yano, Shannon K. Esher, Emily Hardie, Paul L. Fidel, Mairi C. Noverr

**Affiliations:** a Center of Excellence in Oral and Craniofacial Biology, Louisiana State University Health Sciences Center School of Dentistry, New Orleans, Louisiana, USA; University of California, Davis

**Keywords:** *Candida*, intra-abdominal infection, polymicrobial infection, sepsis, trained innate immunity

## Abstract

Polymicrobial intra-abdominal infections (IAI) are clinically prevalent and cause significant morbidity and mortality, especially those involving fungi. Our laboratory developed a mouse model of polymicrobial IAI and demonstrated that coinfection with Candida albicans and Staphylococcus aureus (C. albicans/S. aureus) results in 80 to 90% mortality in 48 to 72 h due to robust local and systemic inflammation. Surprisingly, inoculation with Candida dubliniensis and S. aureus resulted in minimal mortality, and rechallenge of mice with lethal C. albicans/S. aureus conferred >90% protection up to 60 days postinoculation. Protection was mediated by Gr-1^+^ polymorphonuclear leukocytes, indicating a novel form of trained innate immunity (TII). The purpose of this study was to determine the microbial requirements and spectrum of innate-mediated protection. In addition to Candida dubliniensis, several other low-virulence *Candida* species (C. glabrata, C. auris, and C. albicans *efg1*Δ/Δ *cph1*Δ/Δ) and Saccharomyces cerevisiae conferred significant protection with or without S. aureus. For C. dubliniensis-mediated protection, hyphal formation was not required, with protection conferred as early as 7 days after primary challenge but not at 120 days, and also following multiple lethal C. albicans/S. aureus rechallenges. This protection also extended to a lethal intravenous (i.v.) C. albicans challenge but had no effect in the C. albicans vaginitis model. Finally, studies revealed the ability of the low-virulence *Candida* species that conferred protection to invade the bone marrow by 24 h post-primary challenge, with a positive correlation between femoral bone marrow fungal infiltration at 48 h and protection upon rechallenge. These results support and further extend the characterization of this novel TII in protection against lethal fungal-bacterial IAI and sepsis.

## Abstract (precis)

Polymicrobial intra-abdominal infections (IAI) are clinically prevalent and cause significant morbidity and mortality, especially those involving fungi. Our laboratory developed a mouse model of polymicrobial IAI and demonstrated that coinfection with Candida albicans and Staphylococcus aureus (C. albicans/S. aureus) results in 80 to 90% mortality in 48 to 72 h due to robust local and systemic inflammation.

## INTRODUCTION

Intra-abdominal infections (IAI) can occur as a result of bowel perforations, laparotomy surgery, intestinal hernias, and insertion of medical devices, such as peritoneal catheters (1, 2). If these infections are left untreated or misdiagnosed, microorganisms can migrate into the bloodstream, causing sepsis and leading to significant morbidity and mortality (3–5). IAI are often polymicrobial, and infections involving both fungal and bacterial pathogens result in significantly higher mortality rates than infections involving bacterial species only (6–12). Along with Gram-negative enteric bacteria, Gram-positive species including Staphylococcus aureus are also frequently coisolated pathogens, particularly with nosocomial infections (13–18). This polymicrobial pairing can be enhanced based on a predilection of S. aureus for Candida albicans hyphae, which are observed coassociated within peritoneal tissue lesions (19). The pathogenesis of this lethal polymicrobial IAI is not well understood although inflammatory responses leading to sepsis are considered to play a major role (20, 21).

Our laboratory has been studying polymicrobial IAI using coinfection with C. albicans and S. aureus (C. albicans/S. aureus) in an experimental mouse model which results in 80 to 90% mortality by 48 to 72 h postinoculation (22–24). Characterization of host responses during C. albicans/S. aureus polymicrobial IAI demonstrated that mortality is associated with robust inflammation and with elevated levels of hallmark sepsis proinflammatory cytokines (interleukin-6 [IL-6], tumor necrosis factor alpha [TNF-α], and IL-1β), both locally and systemically, as early as 4 h and continuing through 24 to 48 h postinoculation. On the other hand, there were equivalent microbial burdens in nonlethal monomicrobial and lethal polymicrobial infections, both locally in the peritoneal cavity and in adjacent (spleen, kidney) and nonadjacent (brain) organs at similar time points, indicating that robust inflammation (sepsis) rather than microbial burden is the main driver of lethality (23).

IAI studies using non-*albicans Candida* (NAC) species resulted in various levels of mortality. Coinfections with Candida glabrata or Candida dubliniensis and S. aureus showed no mortality, whereas coinfections with Candida krusei or Candida tropicalis and S. aureus resulted in 80 to 90% mortality (25). In all cases, monomicrobial infections with the various fungal species or S. aureus alone were not lethal (24). In subsequent studies, animals given a C. dubliniensis/S. aureus coinfection or C. dubliniensis alone were highly protected (80 to 90%) against a lethal challenge with C. albicans/S. aureus given 14 days later (25). This protection was found to be long-lived (up to 60 days post-C. dubliniensis challenge) but not mediated by adaptive immunity, with protection maintained in RAG^−/−^ mice lacking T and B cells. These results suggested that protection may be mediated by trained innate immunity (TII), which is defined as nonspecific memory mediated by innate cells. TII was first described in macrophages “trained” by epigenetic reprogramming leading to enhanced responsiveness to secondary infection (26). However, in our model clodronate-mediated depletion of macrophages and monocytes did not abrogate protection (25). Rather, a large influx of Gr-1^+^ leukocytes as early as 4 h post-lethal challenge in primary-challenged mice and the subsequent abrogation of protection following antibody depletion of Gr-1^+^ cells indicated a novel role for polymorphonuclear leukocytes in mediating this TII. With protection lasting up to 60 days and considering the short life span (24 h) of polymorphonuclear (PMN) cells, these results suggested that the protective Gr-1^+^ cells were putative, long-lived myeloid-derived suppressor cells (MDSC), which have been reported in other models of sepsis (27) and in patients with candidiasis (28). The purpose of the present study was to further interrogate this novel form of TII by examining the microbial requirements and spectrum of the protective response.

## RESULTS

### Spectrum and requirements of trained innate immune protection mediated by low-virulence fungal species.

**FIG 1:**
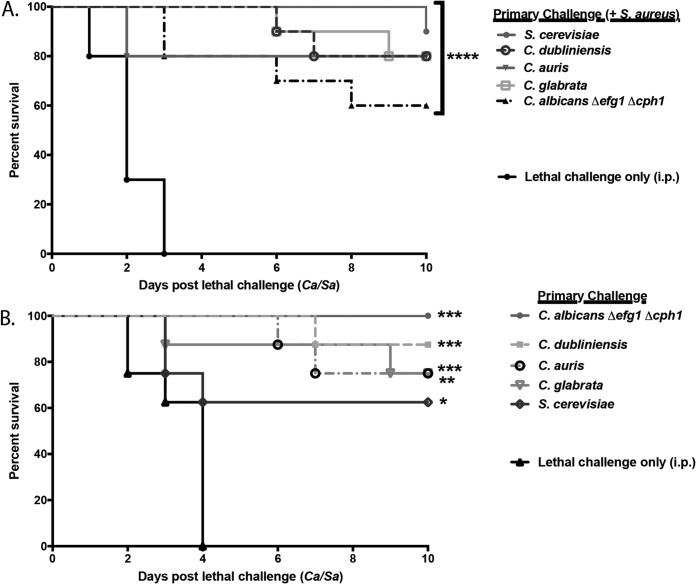
Low-virulence *Candida* species-mediated trained innate immune protection against lethal polymicrobial IAI. Mice (*n* = 10/group) were given a primary i.p. challenge of C. dubliniensis strain Wü284, a C. glabrata clinical isolate, C. auris strain AR0386, C. albicans *efg1*Δ/Δ *cph1*Δ/Δ (DAY185 background), or S. cerevisiae strain FY4 in combination with S. aureus strain NRS383 (A) or alone (B), followed by a lethal challenge of C. albicans/S. aureus (*Ca*/*Sa*) after 14 days. Animals receiving no primary challenge served as the positive (lethal) control. Mice were monitored for 10 days post-lethal challenge. Data are cumulative of three separate experiments. *, *P* < 0.05; **, *P* < 0.01; ***, *P* < 0.001; ****, *P* < 0.0001 (for values significantly different from those of the control, by log rank Mantel-Cox test).

To build upon the initial observation that C. dubliniensis, C. dubliniensis/S. aureus, or C. glabrata/S. aureus primary challenge could confer protection against lethal C. albicans/S. aureus challenge (25), we sought to determine whether other low-virulence *Candida* or non-*Candida* species could also confer protection with or without S. aureus. For studies with S. aureus, groups of mice were given a polymicrobial primary challenge of fungal species including Saccharomyces cerevisiae, C. auris, C. dubliniensis, C. glabrata, or a C. albicans *efg1*Δ/Δ *cph1*Δ/Δ double-null mutant (avirulent C. albicans mutant that is defective in hyphal formation; parental strain SC5314) together with S. aureus, followed by a lethal challenge of C. albicans/S. aureus after 14 days. The survival results are shown in Fig. 1A. Compared to protection conferred by controls that received the C. albicans/S. aureus lethal challenge only, each fungal species tested in combination with S. aureus conferred significant protection against C. albicans/S. aureus lethal challenge (60 to 90%; *P* < 0.0001). Similar results were observed with the monomicrobial primary challenge of each species, albeit at various levels of significance (Fig. 1B).

### Requirement for hyphae in C. dubliniensis-mediated trained innate immune protection.

**FIG 2:**
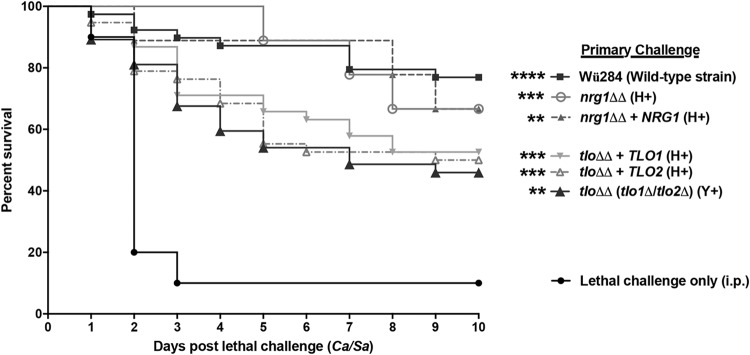
Role of hyphae in C. dubliniensis-mediated trained innate immune protection against lethal polymicrobial IAI. Mice (*n* = 10/group) were given a primary i.p. challenge of wild-type C. dubliniensis Wü284, the *nrg1*Δ/Δ mutant strain, the *nrg1*Δ/Δ *NRG1* reintegrant strain, the *tlo1*Δ/Δ *tlo2*Δ/Δ (*tlo*ΔΔ) double-null mutant strain, or corresponding reintegrant strain (*tlo*ΔΔ *TLO1* or *tlo*ΔΔ *TLO2* strain), followed by C. albicans/S. aureus lethal challenge after 14 days. Animals receiving no primary challenge served as the positive (lethal) control. Mice were monitored for 10 days post-lethal challenge. Data are cumulative of two separate experiments. **, *P* < 0.01; ***, *P* < 0.001; ****, *P* < 0.0001 (for values significantly different from those of the control, by log rank Mantel-Cox test). H+, hyphae; Y+, yeast only.

To determine the role of hyphal formation in C. dubliniensis-mediated protection, we tested several genetically engineered strains with targeted deletions in genes required for morphogenesis (*TLO1, TLO2*, and *NRG1*), complemented mutants (reintegrants), or the parental strain (C. dubliniensis Wü284) as the primary challenge, followed by lethal C. albicans/S. aureus challenge after 14 days. Compared to the level of protection of controls that received the C. albicans/S. aureus lethal challenge only, each mutant induced a significant level of protection, comparable to that of the parental strain Wü284 that conferred ∼75% protection (*P* < 0.0001) (Fig. 2). The *nrg1*Δ/Δ null mutant strain and its reintegrant, the *nrg1*Δ/Δ *NRG1* strain, both of which form hyphae, showed ∼70% protection (*P* = 0.001 and *P* = 0.0026, respectively). The *tlo1*Δ/Δ *tlo2*Δ/Δ double-null mutant (*tlo*ΔΔ strain), which does not form hyphae, and the corresponding reintegrant *tlo*ΔΔ *TLO1* and *tlo*ΔΔ *TLO2* strains, both of which are hyphal forming, all conferred ∼50% protection (*P* < 0.01).

### Spectrum of C. dubliniensis-mediated trained innate immune protection.

**FIG 3:**
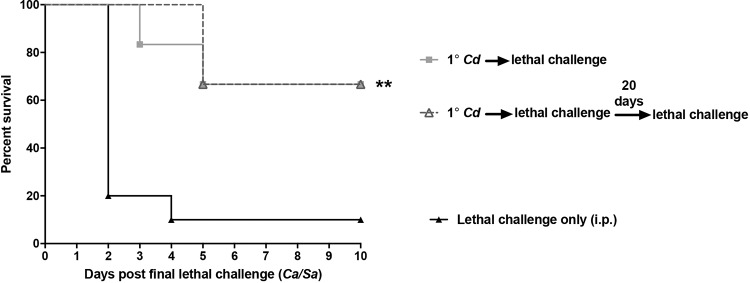
Trained innate immune protection following multiple lethal challenges. Mice (*n* = 10/group) were given a primary i.p. challenge of C. dubliniensis (*Cd*) followed by a single C. albicans/S. aureus (*Ca*/*Cs*) lethal challenge after 14 days or by two consecutive lethal challenges separated by 20 days (for those that survived the first lethal challenge). Animals receiving no primary challenge served as the positive (lethal) control. Each group was observed for morbidity and mortality for 10 days after the final lethal challenge. Data are cumulative of two separate experiments. **, *P* < 0.01 (for values significantly different from those of the control, by log rank Mantel-Cox test).

**(i) Multiple lethal challenges.** To further define the limits of C. dubliniensis-mediated protection, animals given a C. dubliniensis primary challenge (at −14 days) were subsequently given either a single lethal rechallenge or a series of two lethal rechallenges separated by 20 days. Results in Fig. 3 show no difference in survival rates (∼90%) between animals given the single lethal challenge and those that received two lethal challenges (*P* < 0.01).

**FIG 4:**
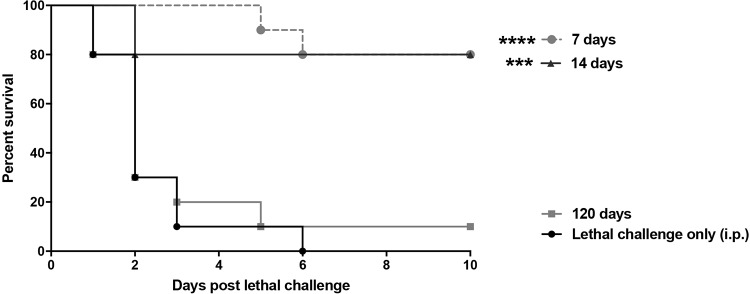
Longevity of protection. Mice (*n* = 10/group) were given a primary i.p. challenge of C. dubliniensis 7, 14, or 120 days prior to C. albicans/S. aureus lethal challenge. Animals receiving no primary challenge served as the positive (lethal) control. Mice were monitored for 10 days post-lethal challenge. Data are cumulative of two separate experiments. ***, *P* < 0.001; ****, *P* < 0.0001 (for values significantly different from those of the control, by log rank Mantel-Cox test).

**(ii) Longevity of protection.** To determine if the protection conferred by C. dubliniensis could be achieved outside the previously reported 14- to 60-day period prior to lethal challenge (25), groups of mice were given the lethal challenge as early as 7 days or up to 120 days following primary challenge. Results in Fig. 4 show that high-level of protection (75 to 80%) compared to that of the control was achieved in mice given the lethal challenge as early as 7 days after primary challenge (*P* < 0.001). However, animals given the lethal challenge 120 days following primary challenge were not protected. In studies to assess the microbial clearance, primary C. dubliniensis-challenged animals had minimal numbers of CFU detected in both the spleen (∼3 × 10^2^ cells/organ) and peritoneal lavage fluid (∼2.5 × 10^1^ cells/ml) after 7 days, and CFU were undetectable at 14 days (data not shown).

**FIG 5:**
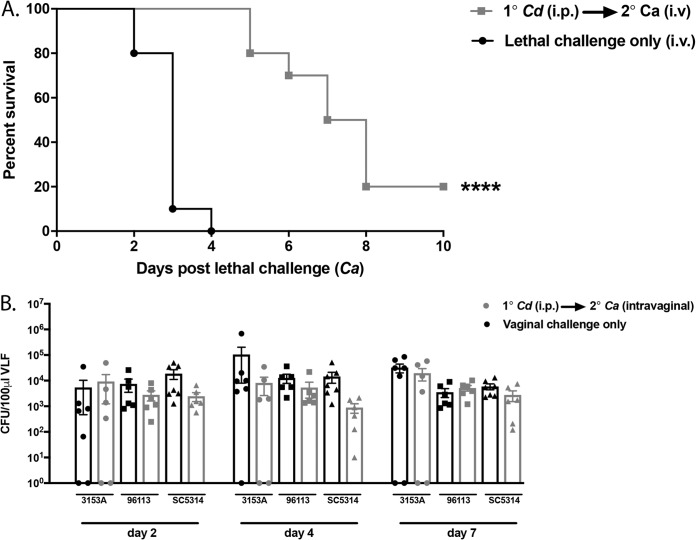
Other models of infection. Mice (*n* = 5 to 10/group) were given a primary i.p. challenge of C. dubliniensis (*Cd*) followed by a lethal C. albicans (*Ca*) intravenous challenge (1 × 10^5^ cells/mouse) and observed for morbidity and mortality for 10 days (A) or by a vaginal challenge of C. albicans 3153A (1 × 10^3^), NUM51 (5 × 10^4^), or DAY185 (5 × 10^5^) and assessed for vaginal fungal burden on days 2, 4, and 7 (expressed as the number of CFU/100 μl vaginal lavage fluid [VLF]) (B). Animals receiving no primary challenge served as the positive control. Data are cumulative of two separate experiments. ****, *P* < 0.0001 (for values significantly different from those of the control, by log rank Mantel-Cox test).

**(iii) Other models of infection.** Primary C. dubliniensis-challenged animals were also tested for protection against C. albicans bloodstream (intravenous [i.v.]) and mucosal vaginal infections. For these studies, mice were given an i.v. (1 × 10^5^ cells/mouse) or vaginal challenge (5 × 10^4^ cells/mouse) with C. albicans 14 days after primary intraperitoneal (i.p.) challenge with C. dubliniensis. In the i.v. challenge model, the majority of animals given the lethal i.v. challenge only succumbed by day 3 (median day of survival [MDS], 3 days), whereas all C. dubliniensis-challenged mice survived through day 5 with a gradual increase in mortality through the day 10 endpoint (MDS, 7.5 days) (Fig. 5A) (P < 0.0001). Of note, despite the eventual mortality in vaccinated mice, traditional sepsis markers (i.e., hypothermia, ruffled fur, and hunched posture) were not observed in these animals at the time of ethical sacrifice. Rather, these animals displayed difficulty maneuvering, indicative of encephalitis often observed following low-dose i.v. challenge. In the vaginal infection model, in which we used several C. albicans strains for inoculation and evaluated mice at several time points postinoculation, no modulation of vaginal fungal burden was observed as a result of the primary C. dubliniensis challenge (Fig. 5B). In addition, we observed no attenuation of neutrophil recruitment or lactate dehydrogenase (LDH) levels in vaginal lavage fluid samples, which are hallmarks of symptomatic infection (data not shown).

### Bone marrow infiltration following primary i.p. challenge.

**FIG 6:**
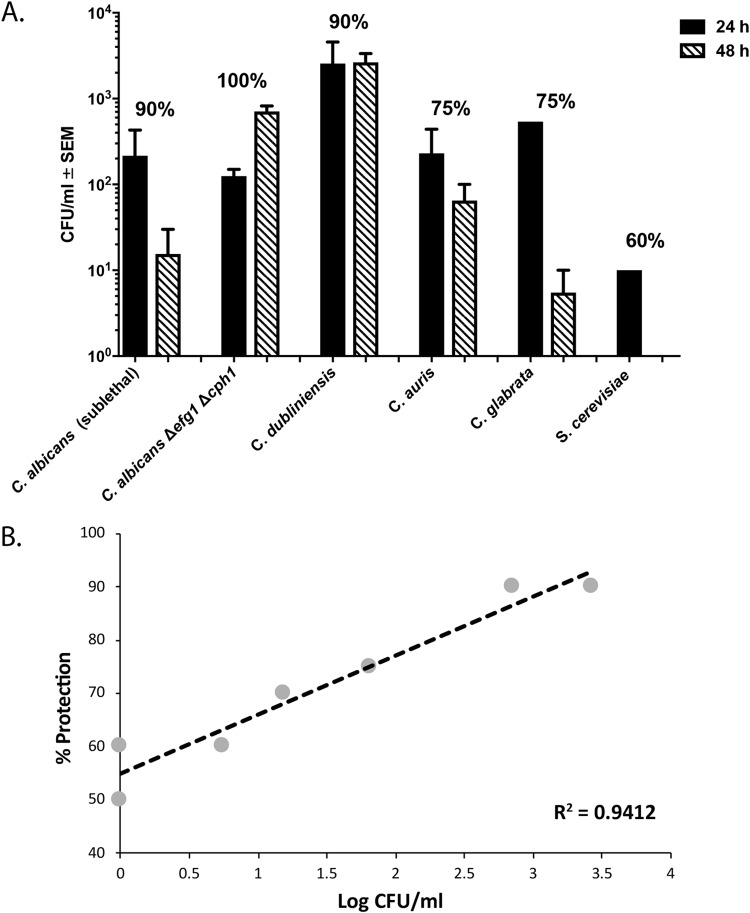
Bone marrow infiltration following primary challenge. (A) Mice (*n* = 2/group) were sacrificed at 24 or 48 h following challenge with C. dubliniensis, C. glabrata, C. auris, C. albicans *efg1*Δ/Δ *cph1*Δ/Δ, S. cerevisiae, or a sublethal inoculum of C. albicans, and femoral bone marrow was isolated and assessed for fungal burden. Results are expressed as the number of CFU/ml of bone marrow cell suspension. Percentages above each set of bars represent protection conferred by each fungal strain upon C. albicans/S. aureus lethal challenge. (B) Correlation (regression analysis) between fungal infiltration into femoral bone marrow at 48 h and the average level of protection recorded for each species in other experiments.

Further interrogation of the events occurring in the mouse that allow for C. dubliniensis-mediated protection in C. albicans/S. aureus IAI began with preliminary investigations to uncover the mechanism of this novel form of TII. Because our previous data suggested that the protection is potentially mediated by MDSC that are largely expanded and activated in the bone marrow (29–31), we evaluated the femoral bone marrow of mice following i.p. primary challenge with the various fungal species described above. Results shown in Fig. 6A revealed that NAC species and S. cerevisiae, as well as C. albicans, could be detected in the femoral bone marrow as early as 24 h postinoculation, with C. dubliniensis-inoculated animals showing the highest fungal infiltration. At 48 h postinoculation the fungal presence persisted at various levels and showed a positive correlation with the average level of protection conferred by these various species (Fig. 6B) (*R*^2^ = 0.9412, *P* < 0.0001). No fungi were detected at 7 or 14 days post-primary challenge. In animals receiving lethal challenge only, detectable levels of both C. albicans and S. aureus were also observed in the bone marrow at 24 and 48 h postchallenge (data not shown).

## DISCUSSION

We have previously reported a convincing role for the induction of protective Gr-1^+^ leukocytes by C. dubliniensis in a novel form of TII against fungal/bacterial IAI/sepsis (25). In this work, we have demonstrated that other low-virulence fungal species can provide various levels of cross-protection against intra-abdominal/systemic infections by multiple species and/or against several rechallenges from a response that is potentially initiated relatively early in the bone marrow and is long-lived. While we previously reported that C. glabrata with S. aureus could also provide strong protection, we were surprised at the number of species that could provide effective protection, including S. cerevisiae. We recognize that only one strain of each species was used and that there could be variation in protection with different strains; however, we previously demonstrated significantly reduced or no protection from several high-virulence species, including C. krusei, C. tropicalis, and even C. albicans (25). Hence, our data suggest that TII is induced at a predictable rate with low-virulence fungal species. Additionally, S. aureus was a relatively minor factor in the overall protection. Results with S. aureus ranged from enhanced protection in the case of S. cerevisiae (suggestive of an adjuvant effect), indifferent effects in combination with C. dubliniensis, C. auris, and C. glabrata, or reduced protection in the case of C. albicans *efg1*Δ/Δ *cph1*Δ/Δ, which was potentially due to increased damage during coinfection. Nevertheless, the high level of cross-protection is further evidence for an innate immune mechanism. This is consistent as well with our previous studies showing that C. dubliniensis/S. aureus primary challenge could provide protection against lethal challenge with C. krusei/S. aureus or C. tropicalis/S. aureus (25).

As mentioned earlier, monomicrobial challenge with more virulent species such as C. krusei or C. tropicalis, as well as with C. albicans, failed to confer protection comparable to that with the low-virulence species (25). This suggests that the damage produced by more virulent species results in less effective induction of TII. This is supported by the differential protection induced by C. albicans versus that with the C. albicans *efg1*Δ/Δ *cph1*Δ/Δ mutant. In alignment with this damage-based hypothesis, it would be interesting to determine the role that candidalysin, a peptide secreted by C. albicans hyphae known to be involved in tissue invasion/damage, plays in protection (32, 33). Another low-virulence *Candida* species was previously reported to provide protection against the more virulent counterpart (via i.v. inoculations) (34). Protection in this case was found to be long-lived (up to 60 days) and mediated by macrophages (based on plastic adherence). This may have represented an early description of what became known as macrophage-mediated TII (26). This finding together with our data further supports a damage-based hypothesis.

In terms of C. dubliniensis-mediated protection, we found no evidence that hyphal formation is required for protection in our IAI model. These results are consistent with our parallel data demonstrating that other non-hyphal-forming species, such as S. cerevisiae, C. auris, C. glabrata, and the C. albicans *efg1*Δ/Δ *cph1*Δ/Δ mutant, can induce significant protection in the absence of hyphal formation. It is important to note that the parental C. dubliniensis strain Wü284, similar to the C. dubliniensis 962926 strain used in our previous studies (25), also provided protection against polymicrobial IAI, providing further evidence for a lack of strain-specific protection. Interestingly, there is also no hyphal requirement for C. albicans/S. aureus synergistic lethality in IAI (23). Together, these data continue to support the concept that the traditional Candida pathogenic mechanism, dependent on the yeast to hypha transition, is not involved in the C. albicans/S. aureus synergistic lethality or the induction of the protective TII response (35, 36).

Perhaps the most significant finding is the infiltration of various fungal species into the bone marrow after a primary i.p. challenge. These findings are more significant for their direct link to MDSC induction and TII than for the observation alone as fungal infiltration into the bone marrow is not often evaluated. We are aware of only one other report demonstrating infiltration of the bone marrow by *Candida* in an experimental infection. That study similarly employed a low-virulence strain of C. albicans that was inoculated i.v. and, through a Toll-like receptor 2 (TLR2)- and dectin-1-dependent mechanism, showed an expansion of hematopoietic stem and progenitor cells (HSPCs) that comprised part of the anti-*Candida* host response (37). Access to the bone marrow has also been reported for mice given the attenuated Mycobacterium bovis bacillus Calmette-Guérin (BCG) vaccine (38). This similarly led to the expansion and reprogramming of HSPCs, which in turn enhanced myelopoiesis. In the case of BCG, the events in the bone marrow eventually give rise to trained innate immune cells (monocytes and macrophages, as well as MDSC) with their own unique epigenetic signatures (38) and the ability to exert protective activity by attenuating T cell-mediated inflammatory responses (39). The BCG vaccine is also known to decrease the morbidity and mortality of other infectious diseases (40, 41), synonymous with a form of TII. These stark similarities to our C. dubliniensis-associated protection further support a trained innate immune mechanism of protection against polymicrobial C. albicans/S. aureus IAI in our model.

Interestingly, the level of bone marrow infiltration of the fungal species tested in our model was correlated with the average level of protection these species conferred. We predict that the strong protection provided by the low-virulence species is reflective of their ability to persist innocuously in the bone marrow and enable the expansion of putative MDSC. For C. dubliniensis, hyphal formation may enhance or accelerate the access to the bone marrow compared to that of the other species. On the other hand, the more virulent C. albicans is predicted to produce more damage in the bone marrow, potentially destroying MDSC precursor cells and/or invoking an innate response that blunts the ability to fully activate MDSC. The use of the candidalysin mutant (C. albicans *ece1*Δ/Δ) may be useful to address these issues further.

The concept that low-virulence species are capable of inducing protective responses with little to no tissue damage parallels the use of a live attenuated vaccine for ultimate induction of adaptive immunity. Of note, the fungal burden was rapidly cleared from both the peritoneal cavity and the spleen of primary-challenged mice (minimal numbers of CFU at day 7 and clearance by day 14), and both C. albicans and S. aureus were completely cleared in protected mice by 20 days post-lethal challenge. Hence, there is a complete resolution of the primary C. dubliniensis challenge without symptoms/morbidity, as well as a complete resolution of the C. albicans/S. aureus infection following lethal challenge in protected mice. These are all excellent properties of an effective live attenuated vaccine that could be considered for more sophisticated vaccine strategies. Protection was induced as early as 7 days and as late as 60 days but not at 120 days post-primary challenge with C. dubliniensis, which reveals the rapid induction, as well as the breadth, of this innate protective response. The significance of the loss of protection at 120 days remains unclear but suggests that protective adaptive immunity is not induced. This may reflect a limit of protection or be due to other factors, such as mouse obesity and/or life span, which should be considered in the interpretations. This limit also alleviates concern over the generation of very long-lived suppressive MDSC that are induced in other models of sepsis and contribute to persistent immunosuppression (42–44). Clinically, this phenomenon is known as compensatory anti-inflammatory response syndrome (CARS) (45, 46), and strategies to either manipulate or inhibit MDSC during CARS are topics of current investigation (42–44).

The extension of C. dubliniensis-mediated protection to other models of *Candida* infection is a highly significant finding with additional translational potential. Although animals did eventually succumb to infection in the C. albicans bloodstream model, significant protection was clearly evident for a period of time before mortality occurred. Furthermore, the mice displayed neurological symptoms rather than the standard systemic pattern of sepsis and/or kidney burden/malfunction. It is unclear whether the signs of brain infiltration/morbidity that eventually led to the encephalitis and ethical sacrifice of these mice are indicative of a bolus i.v. inoculum or whether this protection does not extend to the brain. We favor the former since a bolus i.p. inoculum, which also reaches the brain in mice (23), does not show similar neurological symptoms during survival following C. dubliniensis primary challenge. With regard to mucosal infections, no disease modulation was observed against a vaginal *Candida* infection. The hallmark of vaginal *Candida* infection is an immunopathology involving the inability of Gr-1^+^ neutrophils to effectively kill *Candida* in the vaginal environment (47); hence, it is not intuitive that protection/modulation would be achieved despite the involvement of innate immunity. We did not assess protection in a model of oral candidiasis; however, testing protection in this model may be equally challenging because infection is dependent on immunosuppression (48).

In summary, we have characterized the spectrum and identified several microbial requirements of this novel form of TII in the protective response against polymicrobial IAI/sepsis. The ability of multiple fungal species to provide long-lived cross-protection against fungal/bacterial IAI, which extends to lethal C. albicans bloodstream infections, is a highly significant finding with potential for consideration in vaccine strategies. Equally significant is the ability of these fungal species to infiltrate the bone marrow where putative MDSC expansion and activation take place. Current efforts are focused on the cellular and molecular mechanism(s) of TII, including the confirmation of MDSC expansion/activation in the bone marrow and ultimately effector function.

## MATERIALS AND METHODS

### Mice.

Female Swiss Webster mice, 5 to 7 weeks of age, were purchased from Charles River Laboratories. Animals were housed and handled according to institutionally recommended guidelines. All experiments involving animals were approved by the Louisiana State University Health Sciences Center (LSUHSC) Institutional Animal Care and Use Committee.

### Strains and growth conditions.

C. albicans strain DAY185, a prototrophic derivative of SC5314, was kindly provided by Aaron Mitchell (Carnegie Melon University, Pittsburgh, PA). The C. albicans *efg1*Δ/Δ *cph1*Δ/Δ mutant strain (parental strain, SC5314-CAI4) was kindly provided by Glen Palmer (University of Tennessee Health Science Center [UTHSC], Memphis, TN). C. albicans strains 3153A and NUM51 were obtained from the American Type Culture Collection (ATCC 28367 and ATCC 96113, respectively). C. glabrata was obtained from the Fidel laboratory bank of isolates (LSUHSC, New Orleans, LA). C. auris strain AR0386 was kindly provided by Jose Vazquez (Augusta University, Medical College of Georgia, Augusta, GA). S. cerevisiae, strain FY4, was kindly provided by Fred Winston (Harvard University, Cambridge, MA). The C. dubliniensis wild-type parental strain (Wü284), the genetically engineered gene deletion mutant *nrg1*Δ/Δ strain, the reintegrant *nrg1*Δ/Δ (p*CdNRG1*) strain (expressing the C. dubliniensis *NRG1* gene [*CdNRG1*]) (49), the *tlo1*Δ/Δ *tlo2*Δ/Δ double-null mutant (*tlo*ΔΔ), and the reintegrant *tlo*ΔΔ *TLO1* and *tlo*ΔΔ *TLO2* strains (50) were kindly provided by Gary Moran (Trinity College, Dublin, Ireland). Frozen stocks were maintained at −80°C and streaked onto yeast peptone dextrose (YPD) agar or CHROMagar Candida (CHROMagar, Paris, France) prior to use. A single colony was transferred to 10 ml of YPD broth and shaken at 30°C for 12 to 18 h. The methicillin-resistant S. aureus strain NRS383 used in all experiments was obtained from the Network on Antimicrobial Resistance in S. aureus (NARSA) data bank. Frozen stocks were maintained at −80°C and streaked onto Trypticase soy agar (TSA) prior to use. A single colony was transferred to 10 ml of Trypticase soy broth (TSB) and shaken at 37°C overnight. On the following day, the overnight culture was diluted 1:100 in fresh TSB and shaken at 37°C for 3 h until the culture reached the log phase of growth. Prior to inoculation, both organisms were washed three times by centrifugation in sterile phosphate-buffered saline (PBS; pH 7.4), counted on a hemocytometer, and diluted in sterile PBS to prepare standardized inocula.

### Fungal-bacterial intra-abdominal infection model.

(i) Primary challenge. For most studies, groups (*n* = 10) of 6-week-old outbred Swiss Webster mice were injected intraperitoneally (i.p.) with various *Candida* species (1.75 × 10^7^ cells/mouse) alone or in combination with S. aureus (8 × 10^7^ cells/mouse), in a volume of 200 μl 14 days prior to lethal challenge. In specific experiments mice were given the lethal challenge as early as 7 days or as late as 120 days after the primary challenge or a sublethal C. albicans DAY185 inoculum (7 × 10^6^ cells/mouse). For additional analyses, a subset of the mice were sacrificed at 7 and 14 days after primary i.p. C. dubliniensis challenge, and peritoneal lavage fluid and spleens were collected to monitor local and intraperitoneal/bloodstream microbial burden, respectively, as previously described (24). Briefly, peritoneal cavities were injected with 2 ml of sterile saline, followed by gentle massage of the peritoneal cavity and collection of fluid using a pipette inserted into a small incision in the abdominal cavity. Spleens were removed and mechanically homogenized in sterile PBS. (ii) Lethal challenge. Mice were injected i.p. with a lethal challenge of C. albicans DAY185 (1.75 × 10^7^ cells/mouse) and S. aureus (8 × 10^7^ cells/mouse) in a volume of 200 μl and observed for morbidity (hunched posture, inactivity, and ruffled fur) and mortality up to 10 days after rechallenge. For some studies, survivors of the lethal challenge were given a second lethal challenge 20 days after the previous lethal challenge and observed for morbidity and mortality for an additional 10 days.

### Bone marrow cell isolation.

Mice were sacrificed at 24 or 48 h post-primary challenge or following lethal challenge, and bone marrow was isolated for CFU enumeration. Murine bone marrow was isolated and prepared as previously described (51). Briefly, femurs were isolated from mice, and each bone was flushed with 5 to 10 ml of cold PBS using a 27.5- gauge needle. Red blood cells (RBCs) were lysed in 1× RBC lysis buffer (ThermoFisher), and cells were resuspended in 1 ml of sterile PBS.

### CFU analysis.

Fungal burden in bone marrow-isolated cells, spleen homogenate, and peritoneal lavage fluid was enumerated by plating serial dilutions onto YPD agar containing 20 μg/ml nafcillin and 2 μg/ml vancomycin via the drop plate method (52). For animals receiving the lethal challenge, bacterial burden was also assessed using TSA containing 20 μg/ml nafcillin and 2.5 μg/ml amphotericin B. Plates were incubated overnight at 37°C. CFU counts are expressed as the number of CFU/milliliter of bone marrow cell suspensions, spleen homogenates, or peritoneal lavage fluid.

### Bloodstream infection model.

Mice were given a lethal intravenous challenge of C. albicans DAY185 (1 × 10^5^ cells/mouse) via tail vein injection (100 μl) 14 days after primary i.p. challenge with C. dubliniensis. Mice were observed for morbidity (hunched posture, inactivity, and ruffled fur) and mortality up to 10 days after lethal intravenous challenge. Control mice received the lethal intravenous challenge only.

### Vaginal candidiasis model.

(i) Vaginal inoculation. Vaginal inoculation with C. albicans in mice was conducted as previously described (53). Briefly, mice were administered 0.1 mg of β-estradiol 17-valerate (Sigma, St. Louis, MO) dissolved in 100 μl of sesame oil (Sigma) by subcutaneous injection 72 h prior to vaginal inoculation. Injections were repeated weekly thereafter if required. For the current study, estrogen-treated (estrogenized) mice were intravaginally inoculated by introducing 20 μl of PBS containing C. albicans 3153A (1 × 10^3^), NUM51 (5 × 10^4^), or DAY185 (5 × 10^5^) blastoconidia into the vaginal lumen of naive mice or of mice 14 days after primary i.p. challenge with C. dubliniensis. Groups of 5 mice were evaluated longitudinally for fungal burden. (ii) Vaginal lavage fluid and fungal burden. Under conditions of anesthesia by isoflurane inhalation, vaginal lavage was performed using 100 μl of sterile PBS with gentle aspiration and agitation with a pipette tip as previously described (53). Serial dilutions of the vaginal lavage fluid were cultured on Sabouraud-dextrose agar plates (BD Diagnostics) supplemented with gentamicin (Invitrogen). CFU levels were enumerated after incubation for 24 h at 37°C, and results are expressed as the number of CFU/100 μl of lavage fluid.

### Statistics.

Survival curves were compared using a log rank (Mantel-Cox) test. For longitudinal analyses of vaginal fungal burden, an unpaired Student's *t* test was used with comparisons made between experimental and control groups alone. Significant differences were defined at a confidence level with a *P* value of <0.05. All statistical analyses were performed using Prism software (GraphPad, San Diego, CA).
